# Coinfection of SARS-CoV-2 with other respiratory pathogens in outpatients from Ecuador

**DOI:** 10.3389/fpubh.2023.1264632

**Published:** 2023-10-27

**Authors:** Diana Morales-Jadán, Claire Muslin, Carolina Viteri-Dávila, Barbara Coronel, Bernardo Castro-Rodríguez, Alexander Paolo Vallejo-Janeta, Aquiles Rodrigo Henríquez-Trujillo, Miguel Angel Garcia-Bereguiain, Ismar A. Rivera-Olivero

**Affiliations:** ^1^One Health Research Group, Universidad de Las Américas, Quito, Ecuador; ^2^One Health Research Group, Faculty of Medicine. Universidad de Las Américas, Quito, Ecuador

**Keywords:** coinfection, SARS-CoV-2, outpatients, Latin America, influenza A, Ecuador, *S. pneumoniae*

## Abstract

Worldwide, the COVID-19 pandemic caused by SARS-CoV-2 has enormously impacted healthcare systems, especially in low and middle-income countries. Coinfections with respiratory pathogens in COVID-19 patients may contribute to worse outcomes. This study identified the presence of 12 viral coinfections and pneumococcal carriers among individuals with SARS-CoV-2 infection in outpatient and community settings in Ecuador. From January 2020 to November 2021, 215 nasopharyngeal and nasal swabs were taken from individuals who reported symptoms of COVID-19 or had known exposure to someone with confirmed or suspected COVID-19. One hundred fifty-eight tested positive for SARS-CoV-2 by RT-qPCR and coinfections were detected in 12% (19/158) of SARS-CoV-2-positive patients; the most frequent coinfection was with influenza A virus at 4.4% (7/158; 95% CI: 1.2–7.6), followed by respiratory syncytial virus with 3.1% (5/158; 95% CI: 0.4–5.8), and finally rhinovirus and human coronavirus NL63 with 1.2% (2/158). Pneumococcal carriage was detected in 3.7% (6/158; 95% CI: 0.76–6.64) of SARS-CoV-2 cases. Influenza B, adenovirus, human metapneumovirus (HMPV), parainfluenza virus types 1, 2, and 3, and human coronavirus HKU1 were undetected. To our knowledge, this is the first study of coinfection of SARS-CoV-2 and respiratory pathogens performed on outpatients in Latin America. The high proportion of outpatients with viral coinfections reported in our cohort allows us to suggest that testing for SARS-CoV-2 and other common respiratory pathogens should be carried out to ensure accurate diagnoses, prompt patient treatment, and appropriate isolation.

## 1. Introduction

Coronavirus disease 2019 (COVID-19), caused by severe acute respiratory syndrome coronavirus 2 (SARS-CoV-2), was first reported in China in December 2019 (1). In Ecuador, the first case was confirmed on February 29th, 2020 (2), and between February 2020 and May 2023, more than 732,038 cases and 34,533 deaths associated with SARS-CoV-2 were reported (3).

Patients infected with SARS-CoV-2 commonly develop symptoms 4–12 days after viral exposure. Clinical manifestations range from asymptomatic patients to mild symptoms to severe and critical illness. According to World Health Organization (WHO) clinical criteria for COVID-19 infection, signs or symptoms include fever, cough, fatigue, headache, myalgia, a sore throat, coryza, dyspnea, nausea, and diarrhea (4–6).

Despite the first report from China showed that coinfection with other respiratory pathogens was infrequent, with no other viruses detected and 1% of bacterial coinfection among 99 cases of COVID-19 (7), nowadays, several reports worldwide show evidence that the coinfection rates between SARS-CoV-2 and other viral and bacterial pathogens are higher than initially expected.

In a study conducted in Northern California, Kim et al. found that 20.7% of COVID-19 patients were co-infected with at least one other respiratory pathogen. Respiratory viruses are the most common co-infecting agents (8). In China, 5.8% of patients testing positive for COVID-19 also tested positive for other respiratory viruses (9).

A meta-analysis by Lansbury et al. revealed that common viral coinfections reported in patients positive for SARS-CoV-2 include influenza virus, respiratory syncytial virus (RSV), and adenovirus (10).

In addition, bacterial coinfection is more prevalent among hospitalized patients compared to outpatients, and significantly increases the risk of complications. A nationwide study in Spain performed in 2020 showed that 2.3% of hospitalized COVID-19 patients had bacterial coinfections, which increased to 17% among patients admitted to the intensive care unit (ICU). *Pseudomonas* spp., *H. influenzae* and *S.pneumoniae* were bacteria that caused most infections (11).

Overall, the proportion of bacterial coinfections in SARS-CoV-2 infected patients is estimated to range from 3 to 9% suggesting that bacterial coinfection in patients with COVID-19 is lower than in previous viral pandemics (10, 12–14). During the H1N1 influenza pandemic in 2009, fatal cases were associated with bacterial coinfections, particularly by *S. pneumoniae* (15–17). Reports of coinfection with *S. pneumoniae* during the COVID-19 pandemic range from 0.4 to 11%. Although the lower incidence of bacterial coinfection in COVID-19 patients is not fully understood as well as the impact of *S. pneumoniae* coinfections on COVID-19 severity and the possible interactions between SARS-CoV-2 and *S. pneumoniae* in the nasopharyngeal niche are still being investigated (18–20).

Nevertheless, most of these reports come from Europe, Asia, and the United States, whereas scarce data are available on COVID-19 and respiratory tract coinfections in Latin America. In Peru, two studies detected coinfection with respiratory pathogens in 52.2 and 40.86% of COVID-19 cases, respectively, at hospital admission. The most frequent pathogens were bacteria, while influenza viruses were not detected (21, 22). In Brazil, Pucarelli-Lebrerio et al. reported a 3.5% prevalence of viral coinfection in children diagnosed with COVID-19 infection (23).

The development of molecular tests has improved our ability to detect respiratory pathogens, providing an accurate and more sensitive detection of several pathogens in a faster turnaround time compared to viral and even bacterial cultures. It is a powerful tool because clinically differentiating between an isolated viral infection related to COVID-19 and a possible bacterial or fungal superinfection is challenging (24).

Providing information on both viral and bacterial coinfections associated with community-acquired pneumonia has diagnostic value that may be useful in predicting patient prognosis (8, 25–28) and initiating antiviral therapy (29). Coinfections may have clinical implications associated with increased complications, such as a severe inflammatory process leading to lung damage (30), an extended hospital stay (31), varied treatment approach and duration, and increased mortality rate. Therefore, early detection of coinfection will improve patient management.

In Ecuador, despite the presence of an integrated surveillance plan for COVID-19 and other respiratory viruses established by the National Reference Laboratory of the National Institute for Research and Public Health (INSPI), data on co-infection rates remain inaccessible (32). This study aims to address this gap by determining the prevalence of viral coinfections and pneumococcal carriers detected by molecular methods among outpatients with SARS-CoV-2 infection in Ecuador.

## 2. Materials and methods

### 2.1. Patient recruitment and sample collection

This cross-sectional study describes the prevalence of coinfection with other respiratory pathogens in SARS-CoV-2 infected patients in outpatient and community settings. From January 2020 to November 2021, we evaluated 215 individuals: 121 from hospital outpatient care (San Francisco de Quito, Eugenio Espejo and IESS Sur hospitals) and 94 from primary care settings and/or domiciliary assistance in Quito, Ecuador.

After physician evaluation all patients, regardless of age, who met the inclusion criteria and provided consent, were enrolled in the study, and nasopharyngeal swabs were collected. During the consultation, clinical and sociodemographic data were collected. Comorbidity was categorized according to the International Statistical Classification of Diseases and Problems Related to Health registered by the Pan-American Health Organization (PAHO).

The criteria for inclusion in the study were individuals who had not been hospitalized in the past 15 days at the time of sampling, reported symptoms of COVID-19, or had been exposed to someone with confirmed or suspected COVID-19.

### 2.2. Experimental analysis

All samples were processed in the BSL2-certified molecular biology laboratory at Universidad de Las Américas. Nasopharyngeal and nasal swabs were collected on 0.5 mL TE pH 8 buffer for SARS-CoV-2 diagnosis. All nasal or nasopharyngeal samples were divided into two. The first sample was submitted to a digestion step before DNA extraction and used for identifying *S. pneumoniae*. Nucleic acids were directly extracted from the second sample to detect SARS-CoV-2 and other respiratory viruses.

### 2.3. Digestion and nucleic acid extraction

Nasal and nasopharyngeal swabs were treated by a two-enzyme digestion process to break the *S. pneumoniae* capsule for 8 h before nucleic acid extraction and incubated at 36°C. 100 μL of TE buffer (10 mM Tris–HCl, 1 mM EDTA, pH 8.0) containing 0.04 g/mL lysozyme (Sigma) and 75 U/mL mutanolysin (Sigma) were added to 200 μL of the sample (33, 34). Virus DNA and RNA were simultaneously extracted using Spin Column Extraction Kits (Biocomma Limited, Guangdong, China).

### 2.4. Detection of SARS-CoV-2 by RT-qPCR

The commercial kit ECUGEN SARS-CoV-2 RT-qPCR (Starnewcorp-UDLA, Ecuador) was used to identify the presence of SARS-CoV-2 in nasopharyngeal and nasal swabs. Briefly, the assay is based on The CDC (Centers for Disease Control and Prevention) protocol that includes N1 and N2 probes and RNase P for SARS-CoV-2 detection and RNA extraction quality control, respectively, Freire-Paspuel et al. (35). Also, negative controls (TE pH 8 buffer) were included. A positive control that contains *N* gene of SARS-CoV-2 (IDT, United States) was used for viral load calculation, provided at 200,000 genome equivalents/mL (36–38).

### 2.5. Detection of *Streptococcus pneumoniae* by qPCR

Primers and probes for detecting *S. pneumoniae* have been previously reported (34). The following primers and probes are designed to target the LytA gene and approved by CDC: LytA (forward): ACGCAATCTAGCAGATGAAGCA; LytA (reverse): TCGTGCGTTTTAATTCCAGCT; and probe: 5’-FAM-TGCCGAAAACGC”T”TGATACAGGGAG-3’-SpC “-T” = BHQ1 number access EA005672. In addition, multiplexes designed for real-time PCR proposed by CDC were used for detecting 21 serotypes or serogroups in 7 PCR reactions with protocol triplex sequential real-time PCR-serotyping for Latin America being 14, 18C/18F/18B/18A, 19\u00B0F, 4, 6A/6B/6C/6D, 9 V/9A, 1, 5, 23F, 3, 7F/7A, 19A, 6C/6D, 12F/12A/12B/44/46, 22F/22A, 15A/15F, 23A, 33F/33A/37, 2, 11A/11D and 16F serogroup (39, 40).

### 2.6. Detection of 12 respiratory viruses by multiplex RT-qPCR assays

RNA extracted from nasal and nasopharyngeal swabs was used to synthesize single-stranded cDNAs with the aid of Invitrogen™ Reverse transcriptase SuperScript™ II 200 U/mL, RT Buffer (10X), RT random primers (10x), dNTP (10 mM), RNAse out 40 U/mL, and multiscribe reverse transcriptase.

Four real-time multiplex PCR assays were developed for the detection of 12 respiratory viruses, as shown in Supplementary Table S1 (41–47), including influenza A virus, influenza B virus, rhinovirus, adenovirus, RSV A/B, HMPV, parainfluenza virus types 1, 2, and 3, HCoV types NL63, 229E, and HKU1. The final concentrations of each primer and probe were 500 nM and 300 nM, respectively. Real-time PCR conditions for multiplex 1 and 3 were 95°C for 2 min, followed by 40 cycles of 95°C for 15 s and 64°C for 1 min. Real-time PCR conditions for multiplex 2 and 4 were 95°C for 2 min, followed by 40 cycles of 95°C for 15 s and 60°C for 1 min.

Two synthetic double-stranded DNA fragments were made as positive controls (gBlocks Gene Fragments, IDT). For coronaviruses, the requested DNA sequence contained partial genomic sequences (less than 250 bp in length) of the 3 HCoV types. In the case of respiratory viruses, the requested DNA sequence contains partial genomic sequences (less than 300 bp in length) of 9 human viruses: influenza A, influenza B, rhinovirus 1A, RSV B, HMPV, parainfluenza virus 1, parainfluenza virus 2, parainfluenza virus 3, and human adenovirus C. In the case of *S. pneumoniae*, positive clinical samples were used as controls.

### 2.7. Statistical analysis

The statistical analyses were carried out utilizing SPSS Version 28 for Windows. The continuous variables were expressed as medians and the categorical variables as percentages. The study involved the examination of participants’ characteristics, including age, sex, occupation, symptoms, and comorbidities. The SARS-CoV-2 positive and negative subjects were counted, and the infection status of 13 respiratory pathogens was analyzed in all subjects and grouped to analyze co-infection.

Univariate analysis was conducted using chi-square tests to assess the differences in clinical data between SARS-CoV-2 positive and negative patient groups and between the SARS-CoV-2-only group and the SARS-CoV-2-coinfected group. Odds ratios (OR) and their 95% confidence intervals (CIs) were estimated and statistical significance was set at *p* < 0.05.

Variables with significant *p*-values in the univariate analysis were analyzed with multivariate logistic regression using the stepwise backward Wald method to confirm independence. The Hosmer-Lemeshow test was used to evaluate the goodness of fit of the model.

## 3. Results

This study included 215 nasal and nasopharyngeal swab samples from individuals in outpatient and community settings suspected of having a SARS-CoV-2 infection from January 2020 to November 2021 in Quito, Ecuador. The population’s median age was 32 years, with 122 female and 93 male patients (female/male ratio 1.31). Patients demographics and clinical characteristics are shown in Table 1.

**Table 1:** Demographics and clinical characteristics of all patients included in the study.

		Suspected patients (*n* = 215)	SARS-CoV-2-positive patients (*n* = 158)
Age (years)	Mean (SD)	36.82 (17.9)	37.03 (18.5)
	Range	1–94	1–94
	0–14	21 (9.8%)	16 (10.1%)
	15–24	27 (12.6%)	22 (13.9%)
	25–64	149 (69.3%)	104 (65.8%)
	≥ 65	18 (8.4%)	16 (10.1%)
Sex	Female	122 (56.7%)	92 (58.2%)
	Male	93 (43.3%)	66 (41.7%)
Occupation	Agricultural activities	2 (0.9%)	1 (0.6%)
	Domestic activities	13 (6%)	5 (3.2%)
	Arts	3 (1.4%)	3 (1.9%)
	Natural sciences and mathematics	10 (4.7%)	6 (3.8%)
	Health sciences	28 (13%)	21 (13.3%)
	Commerce	13 (6%)	11 (7%)
	Security corps and protection services	6 (2.8%)	2 (1.3%)
	Education and culture	2 (0.9%)	1 (0.6%)
	Public or private employee	26 (12.1%)	13 (8.2%)
	Student	22 (10.2%)	12 (7.6%)
	Humanities and social sciences	2 (0.9%)	1 (0.6%)
	Trades	4 (1.9%)	2 (1.3%)
	Retired	6 (2.8%)	5 (3.2%)
	Office and administrative work	4 (1.9%)	3 (1.9%)
	Transportation	6 (2.8%)	4 (2.5%)
	Without information	68 (31.6%)	68 (43%)
Comorbidity		36/215 (16.74%)^a^	22/158 (14%)
	Skin and subcutaneous tissue diseases	1 (0.5%)	1 (0.6%)
	Circulatory system diseases	10 (4.7%)	7 (4.4%)
	Connective tissue system disorders	1 (0.5%)	1 (0.6%)
	Digestive system diseases	2 (0.9%)	–
	Respiratory system diseases	3 (1.4%)	2 (1.3%)
	Infectious and parasitic diseases	1 (0.5%)	–
	Endocrine, metabolic, and nutritional diseases	15 (7%)	10 (6.3%)
	Tumors	3 (1.4%)	1 (0.6%)

a Some patients had a combination of chronic medical illnesses: cardiocirculatory + endocrine: 2, cardiocirculatory + genitourinary: 1, cardiocirculatory + respiratory: 1, cardiocirculatory + genitourinary + endocrine: 1, connective system + endocrine: 1.

Overall, 158 patients (73.5%; 158/215) tested positive for SARS-CoV-2 infection in the RT-qPCR assay. Of those, 53.8% (85/158) were from primary care centers and 46.2% (73/158) were outpatients. Fifty-eight percent (92/158) of positive patients were female and 41.7% (66/158) were male. The highest frequency of SARS-CoV-2 infections was in the age group of 25–64 years with 104/158 positives (65.8%).

Of a total of 215 individual enrolled 73% (157/215) reported symptoms and were categorized according to the organ and system affected; all these details are described in Table 2. Several clinical sign and symptom as dysnea, chest pain, anosmia, ageusia, asthenia, arthralgia, and fever were significant more present in patients with SARS-CoV-2 infection than in those without SARS-CoV-2 (*p* < 0.05). The odds ratios of patients with COVID-19 increased by 17.1-fold for asthenia, 12.6-fold for ageusia, 9-fold for arthralgia, and 8.7-fold for anosmia (Table 2). When these factors were analyzed by multivariate logistic regression, only anosmia (odd ratio [OR] 5.23, 95% CI 1.11–24.74) and asthenia (odd ratio [OR]15.25, 95% CI 1.83–127.0) were positively associated to SARS-CoV-2 infection *p* = 0.037 and *p* = 0.012, respectively.

**Table 2:** Descriptive information of sex, comorbidity and symptoms of COVID-19 positive patients.

		SARS-CoV-2-negative *n* = 31 (%)	SARS-CoV-2-positive *n* = 126 (%)	Odds ratios (CI)	*p*-value
Sex	Female	18 (20.2)	71 (79.8%)	1.073 (0.484–2.376)	0.863
	Male	13 (19.1)	55 (43.7%)		
Comorbidity	No	23 (17.6)	108 (82.4)	0.479 (0.186–1.235)	0.122
	Yes	8 (30.8)	18 (69.2)		
Symptoms					
Respiratory	Cough	11 (17.7)	51 (82.3)	1.993 (0.95–4.17)	0.064
	Dyspnea	1 (4.8)	20 (95.2)	8.116 (1–61.9)	0.017*
	Odynophagia	11 (22.4)	38 (77.6)	1.324 (0.624–2.81)	0.463
	Wheezing	0	4 (100)	–	0.225
	Nasal congestion	10 (21.7)	36 (78.3)	1.39 (0.638–3.01)	0.408
Gastrointestinal	Diarrhea	5 (16.7)	25 (83.3)	1.96 (0.71–5.38)	0.188
	Anorexia	0	6 (100)	–	0.136
	Nausea	0	10 (100)	–	0.052
	Vomiting	1 (10)	9 (90)	3.383 (0.419–27.3)	0.226
Musculoskeletal	Myalgia	11 (18)	50 (82)	1.93 (0.925–4.05)	0.076
	Arthralgia	1 (4.3)	22 (95.7)	9.059 (1.2–68.8)	0.011*
	Abdominal pain	0	12 (100)	–	0.032
	Chest pain	1 (5)	19 (95)	7.65 (1–58.56)	0.022*
Sensorineural	Ageusia	1 (3.3)	29 (96.7)	12.6 (1.7–94.7)	0.002*
	Anosmia	2 (5)	38 (95)	8.7 (2.02–37.4)	0.001*
	Conjunctivitis	0	11 (100)	–	0.041
Neurological	Dizziness	1 (25)	3 (75)	1.08 (0.11–10.64)	0.945
	Headache	10 (17.2)	48 (82.8)	2.051 (0.857–7.4)	0.061
Systemic	Fever	5 (12.8)	34 (87.2)	2.85 (1.06–7.7)	0.032*
	Shaking chills	1 (33.3)	2 (66.7)	0.718 (0.06–8.07)	0.787
	Asthenia	1 (2.6)	37 (97.4)	17.1 (2.3–128)	0.000*
	Lymphadenopathy	0	3 (2.4)	–	0.295
Dermatological	Acne	0	3 (100)	–	0.295

^*^ Statistical significance (*p* < 0.05).

Overall, the prevalence of respiratory pathogens other than SARS-CoV-2 in this study was 5.6% (12/215; 95% CI: 2.53–8.67) for influenza A, 4.7% (10/215; 95% CI: 1.87–7.53) for rhinovirus, 2.8% (6/215; 95% CI: 0.59–5.01) for RSV, 0.9% (2/215) for HCoV-NL63, 0.5% (1/215) for HCoV-229E, and 2.8% (6/215) for *S. pneumoniae*. Influenza B, adenovirus, HMPV, parainfluenza 1, 2, and 3, and HCoV-HKU1 were not detected.

The infection rates for influenza A, RSV, HCoV-NL63, *S. pneumoniae* and HCoV 229E do not show significant differences in patients with or without SARS-CoV-2 infection. Only rhinovirus infections were significantly higher in SARS-CoV-2 negative patients (*p* < 0.05) in univariate and multivariate analysis.

Coinfection was detected in 12.02% (19/158; 95% CI: 6.95–17.09) of the SARS-CoV-2 positive cases most of these patients were 25–64 years old (57.9%; 11/19). Fifty-eight point 6 % of them were female and 47.3% were male. Clinical symptoms more present in patients with SARS-CoV-2 coinfection than in those without SARS-CoV-2 coinfection were shaking chills (50%), dizziness (33%), wheezing (25%) and abdominal pain (16.6%).

**Figure 1:**
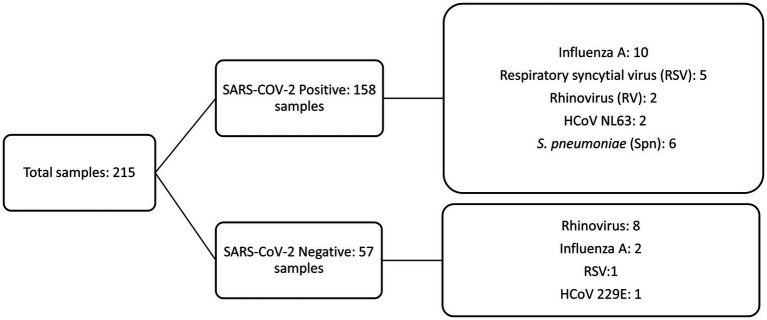
Detection of SARS-CoV-2 infection and coinfection with 13 respiratory pathogens.

Coinfection SARS-CoV-2 with influenza A virus (6.3%, 10/158, 95% CI: 1.2–7.6) was the most frequent followed by RSV with 3.1% (5/158, 95% CI: 0.4–5.8) and finally rhinovirus and HCoV-NL63 with 1.2% (2/158). Regarding pneumococcal carriage, 3.8% (6/158, 95% IC, 0.76–6.64) of the SARS-CoV-2 patients were colonized. Of the 6 patients positive for *S. pneumoniae*, only 5 could be serotyped. Three patients were identified with a single serotype: 3, 6C, and 23F, respectively. The other 2 patients were children of 5 and 6 years colonized with more than one serotype. One patient had serotypes 33A/33F/37, 4, 3, and 18A/18B/18C, and the other had serotypes 3 and 4. Figure 1 summarizes the total number of positive samples for SARS-CoV-2 and presented coinfection.

In two SARS-CoV-2 positive patients, we detected 2 additional pathogens: rhinovirus and *S. pneumoniae* in the first and RSV and *S. pneumoniae* in the second.

Patients coinfected with SARS-CoV-2 and influenza A virus presented significant more wheezing, dizziness and shaking chills (*p* < 0.05). When these factors were analyzed by multivariate logistic regression, wheezing (odd ratio [OR] 10.5, 95% CI 1.01–116) and shaking chill (odd ratio [OR] 15,7, 95% CI 1.25–19.8) keep significant (*p* < 0.05).

**Figure 2:**
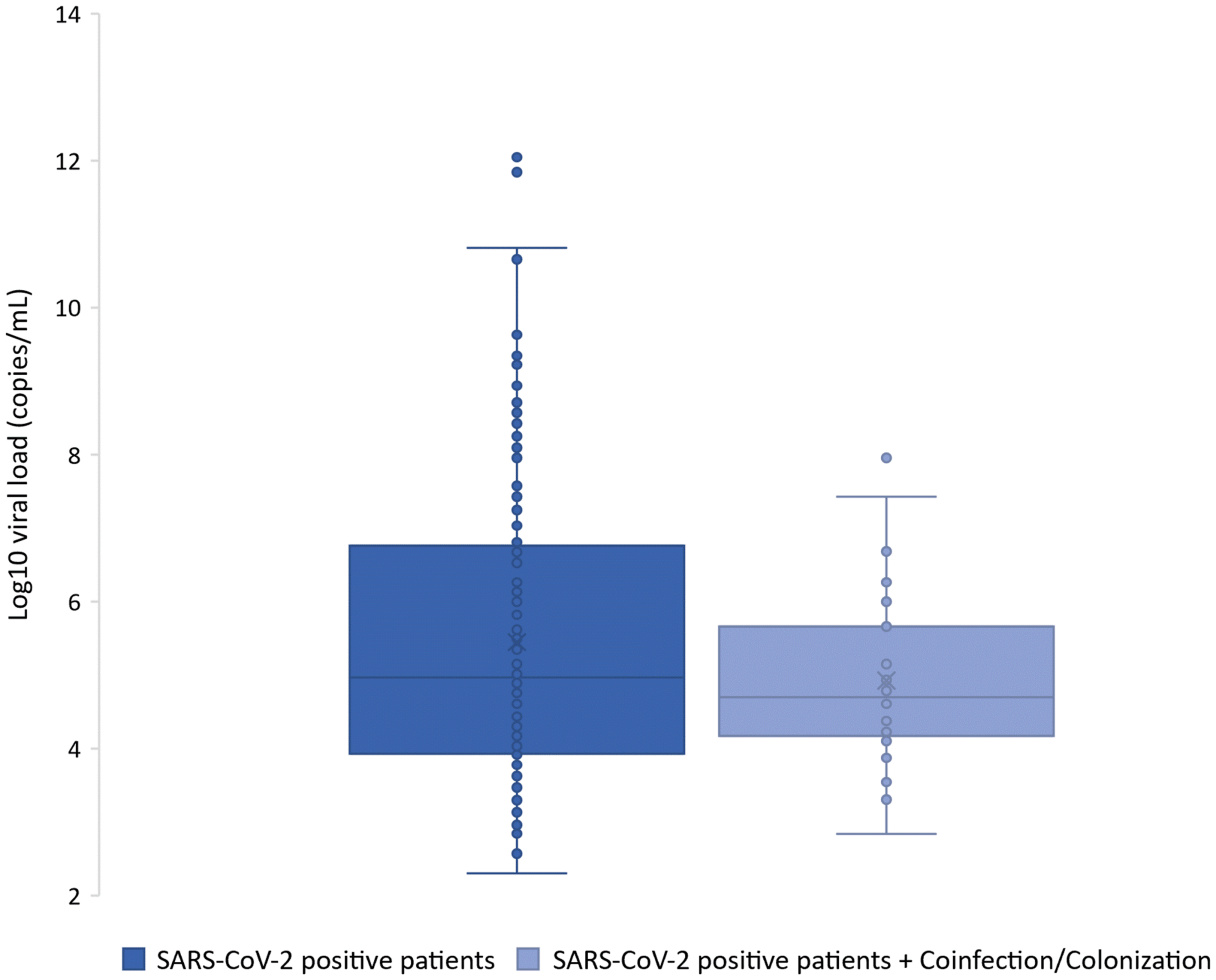
Box plot indicating the log10 of the viral loads in SARS-CoV-2 positive patients and those with coinfection and/or colonization.

The SARS-CoV-2 viral load reported in the 19 coinfected patients had an average of 3.16 × 10^4^ copies/μL for the N1 target. The range was between 1.01 × 10^1^ to 4.48 × 10^5^ copies/μL. Remarkably, all patients with coinfections had a SARS-CoV-2 viral load lower than 5 × 10^5^ copies/μL (Figure 2).

**Figure 3:**
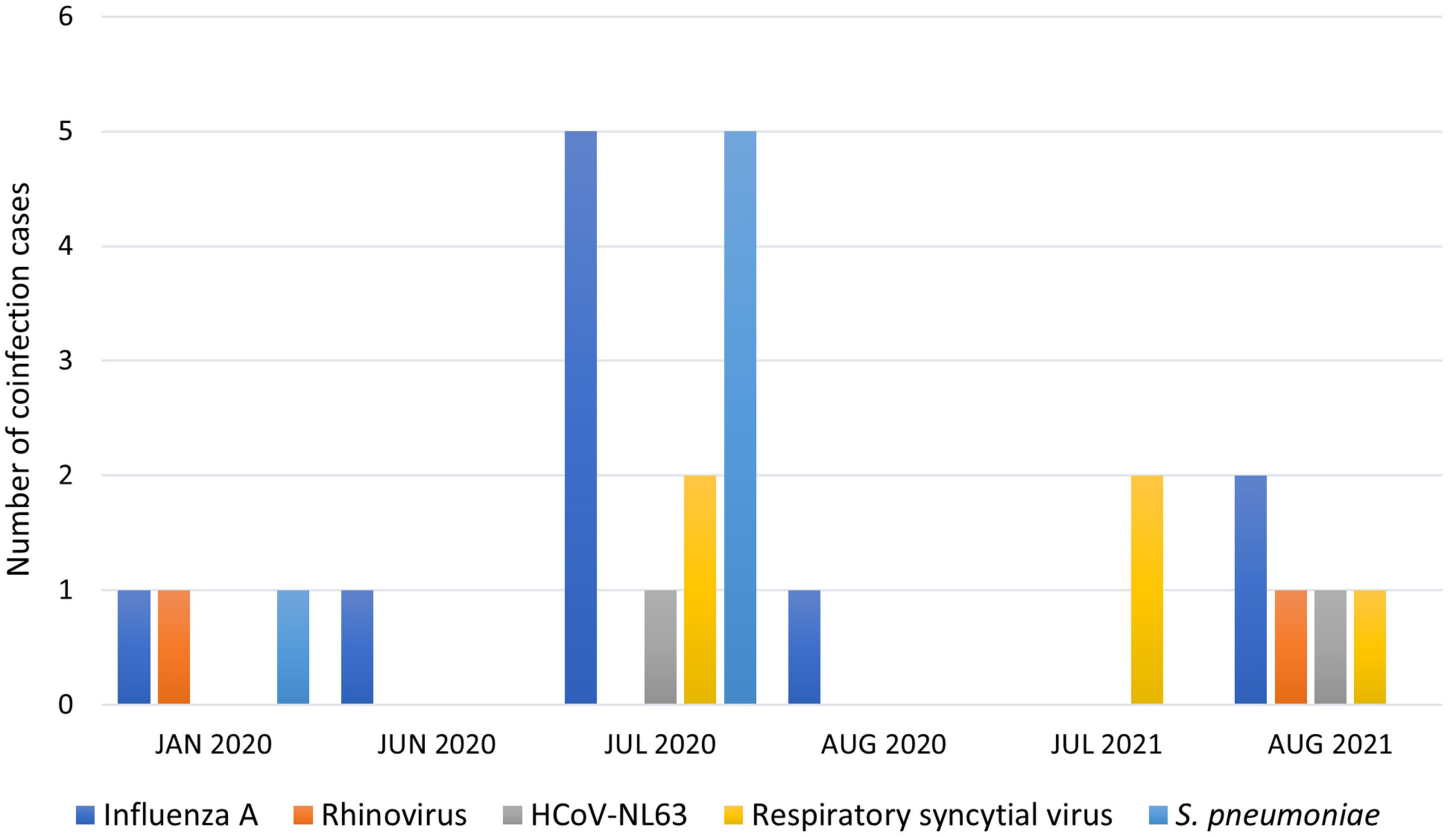
Coinfection cases and date of detection by respiratory pathogens.

All the symptomatic individuals studied had mild illnesses that could be managed in an ambulatory setting or at home through telemedicine. Interestingly, the highest frequency of coinfections was observed within a family group from the south of Quito with 4 cases of SARS-CoV-2 and influenza A, 1 case of SARS-CoV-2 with RSV and *S. pneumoniae*, and 1 case of SARS-CoV-2 with HCoV-NL63, showing a possible household circulation. Also, we observed an increased detection of coinfection from July to August (Figure 3).

## 4. Discussion

The COVID-19 pandemic, caused by SARS-CoV-2, has had a profound impact on global public health, resulting in more than 6.9 million deaths worldwide by April 2023 (48). This pandemic has severely tested the resilience of healthcare systems in low- and middle-income countries, where resources were already limited prior to the outbreak (49, 50). Furthermore, numerous cases of coinfections with respiratory viruses, bacteria, and fungi have been reported in patients infected with SARS-CoV-2 (51, 52).

In Latin America, data on coinfections is scarce. However, in southern Brazil, 19% of patients with SARS-CoV-2 tested positive for rhinovirus, 4.3% for adenovirus, and 1.4% for influenza A (53). In Colombia, Orozco-Hernandez et al. reported a case of SARS-CoV-2 and rhinovirus/enterovirus coinfection in a patient who developed severe respiratory disease and required intensive care unit (ICU) care (54, 55). Additionally, in a Peruvian hospital, 10% of patients with COVID-19 had *Mycoplasma pneumoniae* coinfection (56).

Furthermore, some studies have suggested that coinfections with SARS-CoV-2 and other viruses are less common than bacterial coinfections, particularly with pneumococcus, the most prevalent bacterium in community-acquired pneumonia (39–48).

Coinfection data for COVID-19 are mainly based on hospitalized patients with severe disease. Although most COVID-19 patients do not require hospitalization, very little is known about coinfections among outpatients (57).

This study aimed to fill this knowledge gap by identifying the prevalence of upper respiratory tract coinfections in SARS-CoV-2 positive outpatients. The results showed a 12% prevalence of upper respiratory tract coinfections in SARS-CoV-2 positive outpatients. This study is the first to provide data on the infection rate of SARS-CoV-2, along with other respiratory pathogens, in non-hospitalized patients in Latin America.

Influenza A virus is a common and important coinfecting respiratory pathogen that has been detected in several studies worldwide. Prevalence rates for influenza A virus infection in SARS-CoV-2 positive patients range from 0.08 to 52% in various geographical regions (8, 31, 51, 52, 58, 59). However, each region has a unique profile, and significant variations can exist even within the same country. For example, in China, coinfection rates with influenza A virus have been reported to be as high as 60% in Qingdao, while no coinfection was detected in Wuhan (7, 60). Similarly, in Brazil, coinfection rates with influenza A virus range from 0.04% in Rio de Janeiro to 53% in São Paulo (61). In contrast, in Peru, influenza A virus has not been detected in patients with COVID-19 (21, 22).

Our study found that the prevalence of SARS-CoV-2 co-infection with influenza A virus was 6.3%, followed by RSV at 3.1%, and rhinovirus and HCoV-NL63 at 1.2% among outpatients and the community setting population aged 25–62 years. This suggests the importance of simultaneously screening patients with respiratory tract infections for SARS-CoV-2 and other respiratory viral pathogens.

The prevalence of influenza A virus coinfection in our study was higher than that reported in previous studies of hospitalized patients from Peru and Colombia, where this pathogen was not detected (22, 62). Furthermore, our data showed that patients with influenza co-infection had a higher risk of respiratory symptoms, such as wheezing, and other symptoms, such as dizziness and chills. Coinfection can increase the severity of the disease and the risk of death among high-risk COVID-19 patients, such as the older adult, children, and patients with COPD.

In Ecuador, the high prevalence of SARS-CoV-2/influenza A virus co-infection does not appear to be associated with seasonal influenza, which typically occurs from November to March. Instead, our highest peak of co-infection detection was in July–August. Influenza A virus circulates in Ecuador throughout the year and causes sporadic outbreaks between August and September, though these may occur earlier or later due to various factors (63).

The COVID-19 pandemic has affected the seasonal circulation of many respiratory pathogens, particularly influenza and RSV (64), underscoring the public health risk of co-infection with Influenza A virus and SARS-CoV-2. Therefore, vaccination during seasonal peaks is crucial.

Our study found that RSV was the second most prevalent virus, accounting for 3.1% of coinfections. Worldwide, RSV is a leading cause of bronchitis, bronchiolitis, and viral pneumonia in young, older adult, and immunocompromised patients (65, 66). A report from the UK between February 2020 and December 2021 showed results similar to ours, with a SARS-CoV-2/RSV coinfection rate of approximately 3.2%. Our result was lower than that of a report on hospitalized children in Brazil, which reported a coinfection rate of 18.7% (31, 67).

Regarding pneumococcal colonization, we only detected *S. pneumoniae* in SARS-CoV-2-positive patients. It was the second most frequent pathogen after influenza A virus in our outpatient group, with 3.7% of patients being carriers. These findings are consistent with recent reports in the UK showing that pneumococcal colonization is more frequent among outpatients with mild SARS-CoV-2 infection (34.5%) than in SARS-CoV-2-positive patients (27.4%). This is probably associated with the reduced cellular and mucosal immune responses to SARS-CoV-2 (68, 69). There have been no other studies on pneumococcal colonization in SARS-CoV-2-positive patients in Latin America.

To our knowledge, this is the first study in Latin America to show the prevalence of viral coinfections and *S. pneumoniae* colonization in nonhospitalized SARS-CoV-2-positive patients (outpatients). All symptomatic individuals in our cohort had mild illnesses that could be managed in an ambulatory setting or at home using telemedicine.

The high prevalence of coinfections in this patient group reveals the importance of simultaneous testing for SARS-CoV-2 and other common respiratory pathogens. Molecular screening allows rapid detection, and several commercial panels are available for detecting SARS-CoV-2 and other pathogens in a single reaction to ensure accurate diagnosis, prompt patient treatment, and appropriate isolation, as recommended in several studies (70–74).

Our results must be interpreted in light of our limitations, as this research was restricted to a single region of the country with a limited sample size and could be affected by temporal variation in viral epidemiology. Another limitation of our study is that there was no information about the vaccine status of patients, previous treatment, use of antibiotics, time of disease onset, etc. Moreover, as the study was conducted during the early part of the pandemic and resources were limited, it was not possible to include control group controls to evaluate the presence of asymptomatic infections.

Coinfections in outpatients may lead to changes in the transmission of respiratory pathogens in community settings, allowing pathogens to reach families and community groups and exposing vulnerable populations, such as children and the older adult.

This study provides novel baseline data, highlighting the need for continued surveillance and detection of other pathogens, co-circulation of SARS-CoV-2 and other respiratory pathogens will represent a challenge for health systems globally, but particularly in Latin America, where the health system has not yet fully recovered from the impact of the pandemic.

## Data availability statement

The original contributions presented in the study are included in the article/Supplementary material, further inquiries can be directed to the corresponding author.

## Ethics statement

The study design was approved by the Ethics Committee with code N° 008-2020 and CEISH-HGSF-2021-002 and participants signed a written informed consent after being fully informed.

## Author contributions

DM-J: Formal analysis, Investigation, Writing – original draft. CM: Investigation, Writing – review & editing, Methodology. CV-D: Investigation, Writing – review & editing. BC: Investigation, Writing – review & editing. BC-R: Formal analysis, Writing – review & editing. AV-J: Formal analysis, Writing – review & editing. AH-T: Investigation, Writing – review & editing. MG-B Writing – review & editing. IR-O: Writing – review & editing, Investigation, Methodology, Conceptualization, Supervision.
