# Supplementary material for: Coinfection of SARS-CoV-2 with other respiratory pathogens in outpatients from Ecuador
Source: Front Public Health. 2023 Oct 27;11:1264632. doi: 10.3389/fpubh.2023.1264632 (PMC10641819; doi:10.3389/fpubh.2023.1264632)
Supplement: Supplementary file 1 [file Table_1.docx]

Supplementary Material

Coinfection of SARS-CoV-2 with other respiratory pathogens in outpatients from Ecuador

Diana Morales-Jadan, Claire Muslin, Carolina Viteri-Davila, Barbara Coronel, Bernardo Castro, Alexander Paolo Vallejo-Janeta, Aquiles Rodrigo Henríquez-Trujillo, Miguel Angel Garcia-Bereguiain and Ismar Alejandra Rivera-Olivero*

*** Correspondence:** Rivera-Olivero Ismar Alejandra: iarivera.olivero@gmail.com

**Supplementary table**. Custom primers and probes used for the detection of respiratory viruses

| **Triplex** | **Pathogen** | **Primer sequences (concentration 500nM)** | **Probe sequences (concentration 300 nM)** | **Target** | **Amplicon size** | **Reference** |
| --- | --- | --- | --- | --- | --- | --- |
| **1** | Influenza A Fwd | CATGGARTGGCTAAAGACAAGACC | [6FAM]TGCAGTCCTCGCTCACTGGGCACG[BHQ1] | Matrix protein M1 | 126 | [41] |
|  | Influenza A Rev | AGGGCATTTTGGACAAAKCGTCTA |  |  |  |  |
|  | Influenza B Fwd | TCCTCAACTCACTCTTCGAGCG | [HEX]CCAATTCGAGCAGCTGAAACTGCGGTG[BHQ1] | Nonstructural protein 1 | 103 | [41] |
|  | Influenza B Rev | CGGTGCTCTTGACCAAATTGG |  |  |  |  |
|  | Rhinovirus Fwd 1 | YAGCCTGCGTGGCKGCC | [TexasRed]TCCTCCGGCCCCTGAATGYGGCTAA[BHQ2] | 5¨UTR | 201 | Modified from [42] |
|  | Rhinovirus Fwd 2 | CAGGCTGCGTTGGCGGC |  |  |  |  |
|  | Rhinovirus Rev | GGACACCCAAAGTAGTCGGTRC |  |  |  |  |
| 2 | Adenovirus Fwd | CAGTGGTCTTACATGCACATC | [6FAM]CGAACTGCACCAGMCCSG[BHQ1] | Hexon protein | 132 | Modified from [42] |
|  | Adenovirus Rev | ACSGTGGGGTTTCTAAACTT |  |  |  |  |
|  | RSV-Fwd | GGAAACATACGTGAACAARCTTCA | [HEX]CWGCTGTGTATGTGGAGCCTTCG[BHQ1] | Nucleoprotein | 89 | Modified from [43] |
|  | RSVA-Rev | CATCGTCTTTTTCTARGACATTGTATTGA |  |  |  |  |
|  | RSVB-Rev | TCATCATCTTTTTCTAGAACATTGTACTGA |  |  |  |  |
|  | PIV1-Fwd | GTTGTCAATGTCTTAATTCGTATCAATAATT | [TexaRed]TAGGCCAAAGATTGTTGTCGAGACTATTCCAA[BHQ2] | L polymerase protein | 84 | [44] |
|  | PIV1-Rev | GTAGCCTMCCTTCGGCACCTAA |  |  |  |  |
| 3 | HMPV-Fwd | CATAYAARCATGCTATATTAAAAGAGTCTC | [6FAM]CAACHGCAGTRACACCYTCATCATTRCA[BHQ1] | Nucleoprotein N | 165 | [45] |
|  | HMPV-Rev | CCTATYTCWGCAGCATATTTGTAATCAG |  |  |  |  |
|  | PIV2-Fwd | CCATTTACCTAAGTGATGGAA | AATCGSAAAAGCTGTTCAGTCAC[BHQ1] | hemaglutinin-neuraminidase | 116 | [41] |
|  | PIV2-Rev | CGTGGCATAATCTTCTTTTT |  |  |  |  |
|  | PIV3-Fwd | TTACARATAGGGATAATAACTGT | AAACTCAGACTTGGTACCTGACTTAAAT[BHQ2] | hemaglutinin-neuraminidase | 115 | [41] |
|  | PIV3-Rev | TTAGGAGTGCTAGAGAACAT |  |  |  |  |
| 4 | HCoV-NL63-Fwd | CTCTTTCTCAACCCAGGGCTG | [6FAM]ACCTCGTTGGAAGCGTGTTCCTACCA[BHQ1] | Nucleocapsid | 103 | [46] |
|  | HCoV-NL63-Rev | CGAGGACCAAAGCACTGAATAAC |  |  |  |  |
|  | HCoV-229E-Fwd | CAGTCAAATGGGCTGATGCA | [HEX]CCCTGACGACCACGTTGTGGTTCA[BHQ1] | Nucleocapsid phosphoprotein | 76 | [47] |
|  | HCoV-229E-Rev | AAAGGGCTATAAAGAGARTAAGGTATTCT |  |  |  |  |
|  | HCoV-HKU1-Fwd | CCTTGCGAATGAATGTGCTCAA | [Cy5]ACCACCAGGCTTAACATAATAGCAACCGCC[BHQ2] | orf1ab | 158 | Modified from [46] |
|  | HCoV-HKU1-Rev | GAGAACAAACRTTAGCAGTAACAGC |  |  |  |  |
